# Supplementary material for: Evidence supported by Mendelian randomization: impact on inflammatory factors in knee osteoarthritis
Source: Front Med (Lausanne). 2024 May 28;11:1382836. doi: 10.3389/fmed.2024.1382836 (PMC11165061; doi:10.3389/fmed.2024.1382836)
Supplement: Supplementary file 1 [file Data_Sheet_1.ZIP › data/GCST90274816_5e6/816.forest.pdf]

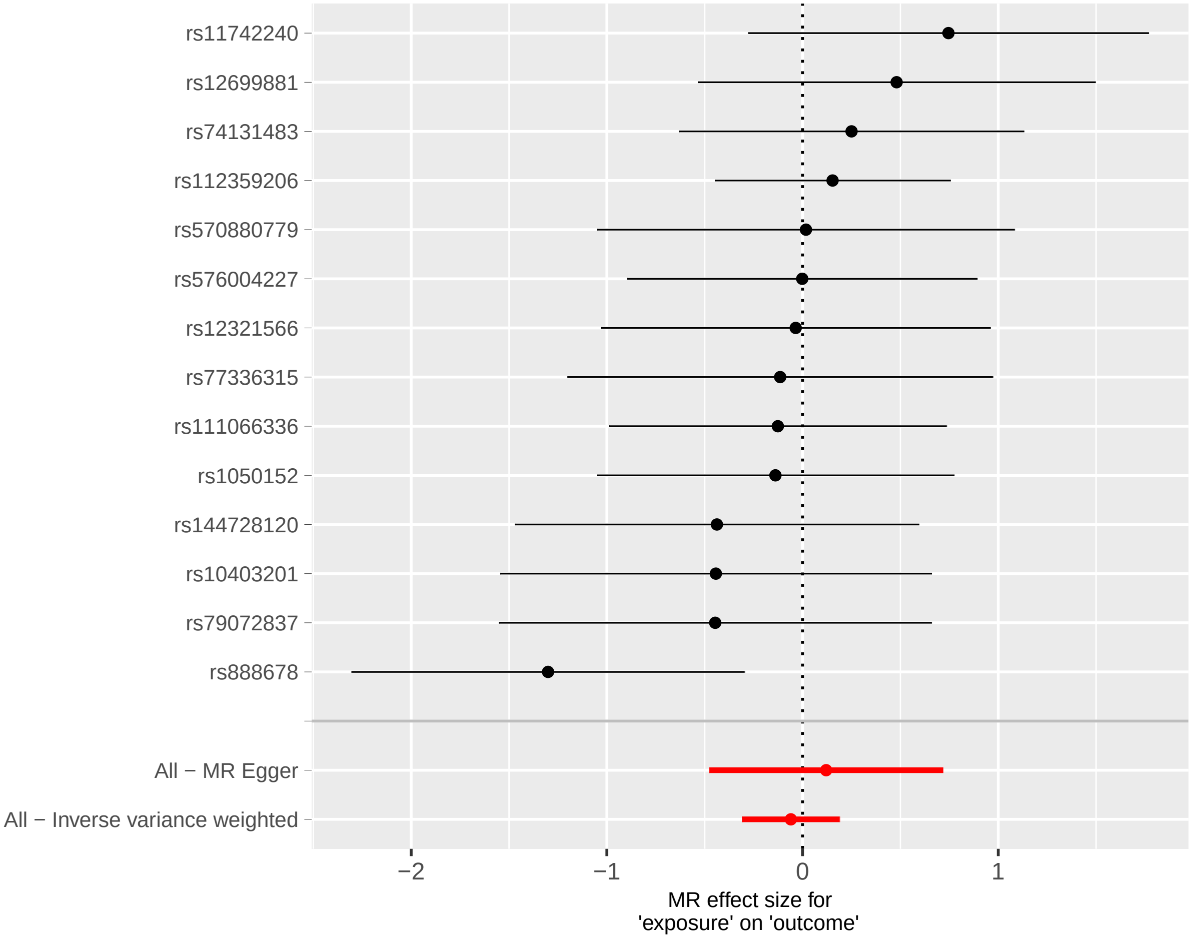
rs11742240
rs12699881
rs74131483
rs112359206
rs570880779
rs576004227
rs12321566
rs77336315
rs111066336
rs1050152
rs144728120
rs10403201
rs79072837
rs888678
All − MR Egger
All − Inverse variance weighted
−2
−1
0
1
MR effect size for
'exposure' on 'outcome'
